# The index herd with PMWS in Sweden: Presence of serum amyloid A, circovirus 2 viral load and antibody levels in healthy and PMWS-affected pigs

**DOI:** 10.1186/1751-0147-51-13

**Published:** 2009-03-27

**Authors:** Per Wallgren, Inger Marit Brunborg, Gunilla Blomqvist, Gunnar Bergström, Frida Wikström, Gordon Allan, Caroline Fossum, Christine Monceyron Jonassen

**Affiliations:** 1 National Veterinary Institute, SVA, 751 89 Uppsala, Sweden; 2 Dept of Clinical Sciences, Swedish University of Agricultural Sciences, SLU, 750 07 Uppsala, Sweden; 3 Section for virology and serology, National Veterinary Institute, Box 8156, Dep 0033 Oslo, Norway; 4 Swedish Animal Health Service, 532 89, Skara, Sweden; 5 Division of Immunology, Department of Biomedical Sciences and Veterinary Public Health, Biomedical Centre, Swedish University of Agricultural Sciences (SLU), Box 588, 751 23 Uppsala, Sweden; 6 Virology Branch, Agri-food and Biosciences Institute, Veterinary Sciences Division, Stormont, Belfast BT4 3SD, UK

## Abstract

**Background:**

Postweaning Multisystemic Wasting Syndrome (PMWS) is an emerging disease in pigs of multifactorial origin, but associated to porcine circovirus type 2 (PCV2) infection. PMWS was first diagnosed in Sweden at a progeny test station that received pigs aged five weeks from 19 different nucleus herds on the day after weaning. The objective of this study was to examine, for the first time in an index outbreak of PMWS, the relationship between PCV2 virus, antibodies to PCV2 and serum amyloid a (SAA) in sequentially collected serum samples from pigs with and without signs of PMWS.

**Methods:**

Forty pigs of the last batch that entered the station at a mean age of 37.5 days were monitored for signs of PMWS during the first 55 days after arrival. Serum was collected on six occasions and analysed for presence of PCV2 DNA and antibodies to PCV2, as well as for levels of SAA.

**Results:**

Four of the pigs (10%) were concluded to have developed PMWS, with necropsy confirmation in three of them. These pigs displayed low levels of maternal antibodies to PCV2, more than 10^7 ^PCV2 viral DNA copies per ml serum and failed to mount a serological response to the virus. Starting between day 23 and 34 after arrival, an increase in PCV2 viral load was seen in all pigs, but PCV2 did not induce any SAA-response. Pigs that remained healthy seroconverted to PCV2 as the viral load was increased, regardless of initially having low or high levels of PCV2-antibodies.

**Conclusion:**

In this index case of PMWS in Sweden, pigs affected by PMWS were not able to mount a relevant serum antibody response which contributed to the disease progression. The maximal PCV2 virus load was significantly higher and was also detected at an earlier stage in PMWS-affected pigs than in healthy pigs. However, a viral load above 10^7 ^PCV2 DNA copies per ml serum was also recorded in 18 out of 34 pigs without any clinical signs of PMWS, suggesting that these pigs were able to initiate a protective immune response to PCV2.

## Introduction

Postweaning multisystemic wasting syndrome (PMWS) is a disease of pigs first recognised in Canada in 1991 that now is a global epizootic [1-3]. PMWS is regarded as a multifactorial disease although infection of pigs with porcine circovirus 2 (PCV2) is recognised as an essential component of the disease process. A difference in pathogenecity between various isolates of PCV2 has been suggested [4-8], but it is also generally accepted that the presence of other infectious or non-infectious factors is required for the development of the full clinical disease [9-12]. Experimental studies in colostrum deprived piglets have demonstrated that such factors can include co-infection with other microbes such as porcine parvovirus [9,10,13], porcine reproductive and respiratory syndrome virus [14,15] or *Mycoplasma hyopneumoniae *[16], but PMWS could also be induced by PCV2 in combinations with either immunsostimulators [17] or immunosuppressors [18]. Experimental infections in both conventional and specific pathogen-free (SPF) pigs with tissue homogenates from PMWS-affected weaners have also induced mild PMWS [19,20]. In these experiments, all inoculated pigs seroconverted to PCV2, but not to any other known virus or bacteria. Transmission of PMWS has also been demonstrated by mixing healthy weaners with PMWS-affected pigs in previously emptied and cleaned facilities [21]. However, the reasons why some pigs develop PMWS while other pen mates remain healthy is still not clear [12,22].

PMWS was diagnosed for the first time in Sweden at a progeny test station in December 2003 [23]. As a consequence the station was closed down, but all pigs present at the station were reared to the weight of 100 kg before closure. To date there have been no reports in the literature on the investigation of the health status related to the load of PCV2 in blood, the level of antibodies to PCV2 virus and serum amyloid A (SAA) determined in sequentially collected serum samples from an on-going index case of PMWS. Within the last batch of pigs reared at the test station, this was determined in 40 pigs that also were monitored closely for clinical signs of PMWS.

## Methods

### Initial health status of the animals

Pigs in Sweden are free from all diseases listed by the Office International des Epizooties (OIE), including Aujeszky's disease (AD), porcine reproductive and respiratory syndrome (PRRS), and also from porcine endemic diarrhoea (PED) and transmissible gastro-enteritis (TGE). The animals in this study emanated from purebred nucleus herds also declared free from atrophic rhinitis (toxin producing strains of *Pasteurella multocida*), *Salmonella *spp, swine dysentery (*Brachyspira hyodysenteriae*) and mange (*Sarcoptes scabiei*). Infections with *Mycoplasma hyopneumoniae *and *Actinobacillus pleuropneumoniae *are widespread in the conventional pig population in Sweden, but the influence of these diseases has decreased since the 1990s due to the commonly performed age segregated production from birth to slaughter [24].

### Herd, animals and experimental design

The present study that was conducted at a progeny test station was approved by the ethical committee in Uppsala, Sweden (C38/4). The test station was established in March 2002, and introduced intensified rearing strategies previously not used in the country with the aim of improving genetic selection. Briefly, boars from 19 nucleus herds (pure bred Landrace, Yorkshire or Hampshire) were allocated to the test station on the day after weaning at the age of approximately five weeks. On arrival they were mixed with boars of the same age from other herds, and the animals were remixed according to weight every fortnight four times before entering the pen for individual testing. During the individual test period the boars were still group housed, but individually fed via transponders.

In December 2003 PMWS was diagnosed in this herd as the index case of Sweden [23] by employing the internationally accepted criterias for diagnosing PMWS at individual and herd levels [25,26], As a consequence, the station was closed down, but animals already at the station were reared to echo-sounding at market weight before being slaughtered.

The 40 pigs selected for this study belonged to the last group that entered the test station before closing. The pigs that were mixed with each other on arrival came from 10 nucleus herds (Table 1). The health status of the animals was recorded during the first 55 days after arrival. Pigs attended for clinical signs resembling PMWS (*i.e*. underweight or obvious loss of weight) during this time were denoted as "thin". Pigs that died or were euthanized during the observation period were sent for necropsies whenever suitable. The necropsies were carried out at Analycen AB (Lidköping, Sweden), and formalin fixed samples were sent to the National Veterinary Institute SVA for histological and PCV 2 immunohistochemical analyses.

**Table 1:** Herd of origin, breed, mean weight and age of the 40 pigs examined.

							
Herd of origin	Breed			Age Days	Weight Kg	PCV2 antibodies Range	Denoted "thin"
					
	Y	L	H				Not PMWS – PMWS
Y1-SPF	3	-	-	41.3 ± 1.2	11.9 ± 10.6	2.2 – 2.8	1 – 0

Y2-conv	4	-	-	36.5 ± 1.3	10.8 ± 0.9	2.2 – 3.1	1 – 1
Y3-conv	4	-	-	39.0 ± 0.8	10.1 ± 1.6	2.2 – 3.1	0 – 1
Y4-conv	4	-	-	39.5 ± 1.3	13.3 ± 2.0	2.2 – 2.8	-
L1-conv	-	4	-	36.8 ± 2.2	10.8 ± 0.7	2.5 – 3.4	-
L2-conv	-	4	-	33.3 ± 3.3	11.5 ± 1.0	2.2 – 3.1	-
L3-conv	-	4	-	33.5 ± 5.7	10.7 ± 1.9	2.2 – 2.8	0 – 1
L&H-conv	-	3	2	39.8 ± 4.9	11.3 ± 2.1	2.2 – 3.1	-
H1-conv	-	-	4	40.3 ± 2.9	12.1 ± 2.0	2.2 – 3.7	-
H2-conv	-	-	4	35.3 ± 3.7	11.9 ± 1.4	2.5 – 3.1	0 – 1
							
**Overall**	**15**	**15**	**10**			**2.2 – 3.7**	**2 – 4**
**Mean**				**37.5 ± 4.0**	**11.4 ± 1.6**		

Y = Yorkshire, L = Landrace, H = Hampshire; SPF = SPF-herd

The table also shows the Log-range of serum antibody titres to PCV2 when determined at nine days after arrival, the number of pigs denoted as thin or diagnosed with PMWS before day 55 after arrival.

### Collection of blood and analyses performed

Blood samples without additives were collected by jugular vein punctures on days 9, 17, 23, 34, 43 and 55 after arrival. The sera were separated and stored at -20°C until analysed.

Presence of PCV2 in individual serum samples was measured using a quantitative real time PCR assay previously described [27]., with a detection limit of 1,100 DNA copies per ml (Log 3.04). In brief, nucleic acids were extracted from 200 μl serum using an EasyMag nucleic acid extractor (Biomerieux, Durha, USA) and eluted in 55 μl elution buffer. For the quantitative PCR, 2.5 μl of each elute was run in a 25 μl reaction with primers and probe previously described [27] on an MxPro 3005 PCR machine (Stratagene, La Jolla, USA). The detection limit of the PCR was 1.1 × 10^3 ^(10 Log 3.04) genome copies per ml serum, and results are presented as 10-logaritms.

Antibodies specific to PCV2 in serum were measured using an immuno-peroxidase-monolayer-assay (IPMA) method previously described. [28]. In brief, freshly trypsinized cells of the PCV-free continuous cell line PK15 A were inoculated with PCV 2 (Stoon-1010) [29]. The inoculated cell-suspension was seeded in 96-well cell culture plates and incubated for 5 days at 37°C (5% CO_2_). The culture medium was removed and the cells were washed with physiological saline. The plates were then fixed in 99.5% ethanol for one hour. The ethanol was removed and glycerol (87%) diluted 1:1 in PBS was added and the plates were kept at -20°C until further use. The glycerol was removed and the plates were washed with PBS containing 0.05% Tween (PBS-T). The serum samples were diluted in PBS-T with 5% fat-free milk powder in a total volume of 100 μl and the plates were incubated for 1 hour at 20°C. After washing with PBS-T the plates were incubated with HRP-conjugated rabbit anti-swine immunoglobulins (DakoCytomation, Glostrup, Denmark) diluted in PBS-T with 5% fat free milk for 1 hour at 20°C. The plates were washed with PBS-T and 50 μl of a substrate solution of 3-amino-9-ethylcarbazole with 0.05% H_2_O_2 _in 0.05 M Na-acetate buffer, pH 5, was added to the wells and the plates were incubated at 20°C for 15 minutes. The reaction was stopped by replacing the substrate with sodium acetate buffer and the results were examined with a microscope.

The antibodies specific to PCV2 were measured in individual serum samples diluted in twofold dilutions from 1:10 to 1:20,480 (Log 1.0 to Log 4.3), The results are presented as Log 10 levels of the antibody titres and seroconversion between two consecutive samplings was defined as an increase with at least two titre steps, corresponding to an increase with at least Log 0.6.

The serum levels of the acute phase protein Serum Amyloid A (SAA) were analysed using a commercial kit (Serum Amyloid A Assay TP-802, Tridelta, Maynooth, Ireland) according to the instructions of the manufacturer. The results are presented as mg SAA per L serum.

### Statistics

All results in the text are given as mean values ± standard deviations. Groups of pigs were compared using Student's t-tests in pair wise comparisons between groups. For comparisons within groups over time, consecutive recordings were compared with each other using paired t-tests.

## Results

The pigs studied were mixed with each other on arrival to the test station at a mean age of 37.5 ± 4.0 days (Table 1). Six of the 40 pigs used in this study were denoted as "thin" during the observation period (Table 2). One of these pigs came from an SPF-herd and was denoted as "thin" on day 12 after arrival at 49 days of age. The other five pigs were recorded as "thin" between day 20 and day 46 after arrival (ranging from 59 to 86 days of age). Two of these six pigs (581-SPF and 1008) were alive at the end of the observation period on day 55. One of the pigs (666) died from wasting on day 39 after arrival, and the remaining three pigs (1037, 842 and 418) were euthanised due to wasting on day 46. PMWS was confirmed as the cause of death in all these three pigs (Table 2) according to international standards [25,26], which apart from wasting included enlarged lymph nodes macroscopically with typical histological lesions and the presence of an abundance of PCV2 antigen in these lesions.

**Table 2:** The six pigs denoted as "thin" within the first 55 days after arrival to the test station.

Pig		Arrival			Denoted as thin			
				
ID	Breed	Age (days)	Weight (kg)	DWG (from birth)	Day after arrival	Age (days)	Status Day 55 after arrival	Diagnose at necropsy
581	Y-SPF	40	11.8	258	12	52	Alive	-
1008	Y	39	9.6	208	20	59	Alive	-
								
666	L	42	9.0	179	20	62	Dead	Not done
1037	Y	36	11.0	264	32	68	Euthanized	PMWS
842	Y	38	11.4	261	41	79	Euthanized	PMWS
418	H	40	13.7	305	46	86	Euthanized	PMWS

Y = Yorkshire, L = Landrace, H = Hampshire; SPF = of SPF-origin

The birth weight was standardised to 1.5 kg when the daily weight gains were calculated

**Figure 1:**
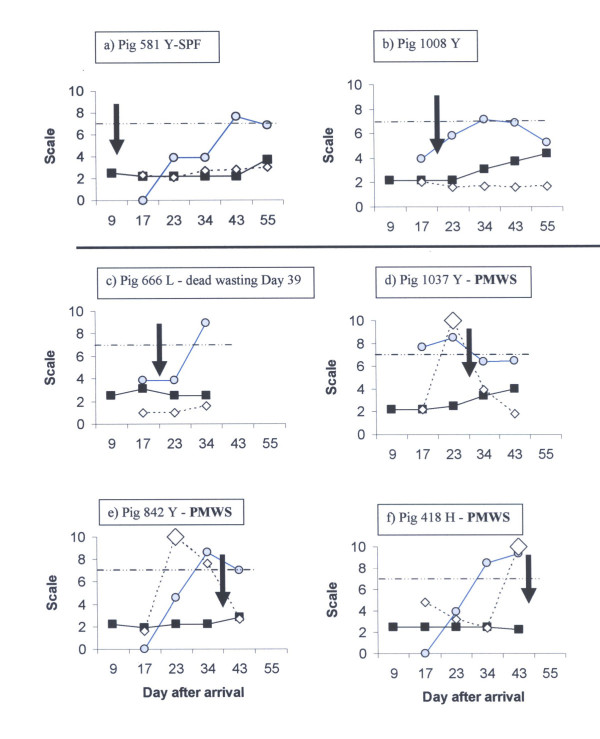
**Individual recordings for pigs attended as "thin"**. The black arrows indicate date when the pig was recorded as "thin" for the first time. The Y-axis shows the PCV2 genome copies per ml serum (grey circles) and serum antibody titres to PCV2 (black squares) as Log 10 values. The serum level of SAA (white diamonds) are presented as mg per L serum × 10. Enlarged white diamonds indicate that the level is above 100 mg SAA per L serum (equal to 10 on that scale). The retarded growth recorded for pigs 581 (a) and 1008 (b) was not accompanied with development of other symptoms of PMWS and these pigs were still alive on day 55. The other four thin pigs got the diagnosis PMWS and were dead on day 55. PMWS was confirmed by necropsy in three euthanized pigs (d-e), whereas necropsy not was performed on pig c that died of wasting.

The serum levels of PCV2 virus DNA, antibodies to PCV2 and SAA of the six pigs denoted as "thin" are shown individually in Figure 1. No increased amounts of PCV2 virus DNA in serum was recorded in SPF-pig 581 or pig 1008 when they were denoted as "thin" on day 12 and day 20 after arrival, respectively. Both these pigs expressed a PCV2 virus DNA load above 10^7 ^at a later stage during the observation period (day 43 and 34 after arrival), and at that time also responded by seroconversion to PCV2 (Figure 1). Thus, these two pigs were not diagnosed as having PMWS, and it is noteworthy that they were the only two pigs denoted as "thin" that were still alive at day 55 after arrival.

In four of the six pigs diagnosed as having PMWS (Table 2), the wasting coincided in time with serum levels of PCV2 exceeding 10^7 ^per ml. None of these pigs showed a clear seroconversion to PCV2 in relation to this increased serum load of PCV2 (Figure 1). As outlined above the remaining two "thin pigs in this group showed an active seroconversion to PCV2. A serum antibody titer of Log 4 was recorded in pig 1037 on day 43, but this pig had been attended as "thin" on day 32, preceded by PCV2 viral loads of 10^7.7 ^and 10^8.5 ^per ml serum on day 17 and 23, respectively, without seroconverting at that time (antibody levels Log 2.2 and Log 2.5, respectively). Pig 1037 was diagnosed with PMWS by necropsy on day 46, but the load of PCV2 had decreased to below 10^7 ^per ml serum at day 43. Thus it cannot be excluded that this pig was in an early phase of recovery from PMWS at the time for necropsy.

**Figure 2:**
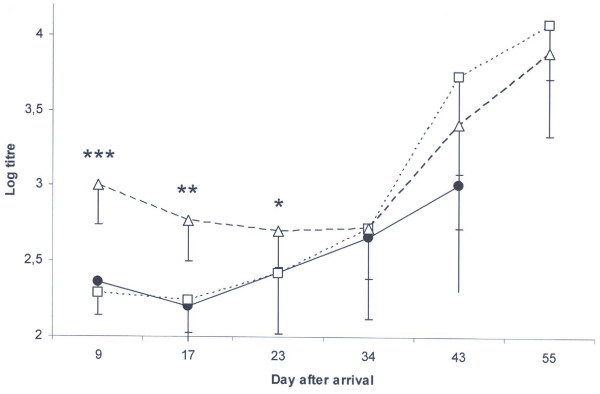
**Mean log titres of antibodies to PCV2 in serum**. Black circles represent pigs diagnosed with PMWS (n = 4). All these pigs were dead at day 55. Pigs without clinical signs of PMWS were grouped as having low (squares, n = 17) or high (triangles, n = 17) maternal immunity to PCV2. All these pigs were alive at day 55. Significant differences between the group with high maternal immunity and the other groups are indicated in the figure (p < 0.05 = *, p < 0.01 = **, p < 0.001 = ***).

As the first sampling occasion occurred nine days after weaning at a mean age of 46.5 ± 4.0 days, the antibody status at that time is referred to as remaining maternal immunity. At that time, the mean serum antibody titre of the six pigs denoted as "thin" ranged from Log 2.2 to Log 2.5 with a mean value of Log 2.36 ± 0.18 for the four pigs diagnosed with PMWS (Figure 1) and of 2.2 for each of the two other pigs. The 34 pigs that remained free from signs of PMWS during this period were divided into two groups according to the level of maternal antibodies to PCV2. One group had a similar range (log 2.2 to 2.5) as the pigs later diagnosed with PMWS with a mean titre of 2.29 ± 0.15 (n = 17), while the other group had higher amounts of maternal antibodies (log 2.8 to 3.7) with a mean titre of 3.00 ± 0.26 (n = 17). Despite decreasing (p < 0.01) from arrival to day 34, the amounts of serum antibodies to PCV2 in the latter group was significantly (p < 0.001 to p < 0.02) higher than in the other two groups until day 23 after arrival (Figure 2).

Increasing (p < 0.01) amounts of antibodies to PCV2 was observed from day 17 after arrival in the group with low amounts of maternal antibodies that remained healthy, and a clear seroconversion (p < 0.001) to PCV2 was observed in both the healthy groups between day 34 and 43 after arrival (Figure 2). In contrast, antibody levels in the four pigs diagnosed with PMWS did not increase (p = 0.22) between days 34 and 43, and all four pigs that developed PMWS were dead on day 55. As seen in figure 1, the two "thin" pigs that survived until slaughter showed a clear seroconversion to PCV2 in relation to increased PCV2 virus levels in serum.

**Figure 3:**
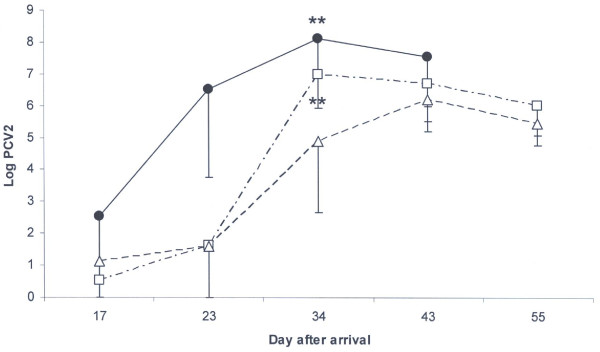
**Mean log levels of PCV2 DNA copy number in serum**. Black circles represent pigs diagnosed with PMWS (n = 4). All these pigs were dead at day 55. Pigs without clinical signs of PMWS were grouped as having low (squares, n = 17) or high (triangles, n = 17) maternal immunity to PCV2. All these pigs were alive at day 55. ** illustrates that the indicated groups differ (p < 0.01) from the group with high maternal immunity to PCV2 at that day.

The amount of PCV2 in serum, measured by PCR detection of nucleic acid increased to Log 6.53 ± 2.77 at day 23 after arrival in the four pigs later to be diagnosed with PMWS (Figure 3). On day 34, both groups with low levels of maternal antibodies had higher (p < 0.005) load of PCV2 in serum than the pigs with high levels of maternal antibodies.

At an individual level, the pigs peaked in PCV2-load in serum on day 23, 34 or 43 after arrival (Table 3). When comparing the peak load of virus regardless of when it took place in time, pigs with the diagnosis PMWS expressed a higher peak viral load than both the other groups (p < 0.001). Furthermore, the healthy pigs with low levels of maternal antibodies to PCV2 peaked with a higher (p < 0.05) viral load than pigs with high levels of maternal antibodies. Pigs with the diagnosis PMWS peaked at 33.5 days after arrival, whereas healthy pigs with high levels of maternal antibodies peaked at day 40 (Table 3).

**Table 3:** Time point and magnitude for the maximal PCV2 loads in serum

Category		Peak	Peak	t-test versus	
			
	N	Day after arrival	Log PCV2	Low	High
PMWS	4	33.5 ± 8.2	8.9 ± 0.4	P < 0.001	p < 0.001
Healthy, low level of maternal antibodies	17	36.7 ± 5.7	7.3 ± 1.0	-	p < 0.05
Healthy, high level of maternal antibodies	17	39.8 ± 9.1	6.5 ± 0.9	p < 0.05	-

**Figure 4:**
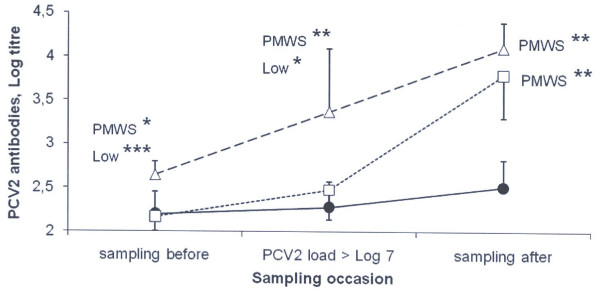
**Increase in serum antibody levels to PCV2 in comparison to when a viral load of 10^7 ^was measured in serum for the first time in pigs of different health and antibody status**. Black circles represent pigs that developed PMWS (n= 4). Pigs without clinical signs of PMWS are grouped as having low (squares, n = 11) or high (triangles, n = 7) maternal immunity to PCV2. Significant differences to other groups are indicated in the figure (p < 0.05 = *, p < 0.01 = **, p < 0.001 = ***).

As shown in figure 4, the serum antibody levels of the four pigs with the diagnosis PMWS was similar when the viral load exceeded 10^7 ^as it was the week before (Log 2.20 ± 0.25 vs Log 2.28 ± 0.29), and it still remained at that level one week later (Log 2.51 ± 0.31). In contrast, pigs with a viral load of PCV2 exceeding 10^7 ^that remained healthy increased (p < 0.05 to 0.001) their antibody levels between the corresponding sampling occasions, regardless of having low (n = 11) or high (n = 7) levels of maternal antibody levels. One week after that the viral load of PCV2 exceeded 10^7^, both these groups differed (p < 0.01) significantly (p < 0.01) to that of the pigs that developed PMWS with respect to level of serum antibodies to PCV2 (Figure 4).

As seen in Figure 1, individual pigs expressed high levels of SAA in serum at different time points, and pigs could have increased levels of serum-PCV2 without a contemporaneous SAA-response and vice versa. With one exception, no significant differences in SAA levels in serum were obtained between the three groups at any occasion (Table 4). The highest level of SAA in serum was obtained at the first sampling occasion at day 9 after arrival. At that time more than 100 mg SAA per L serum was obtained in 10 out of the 40 pigs. However, high levels of SAA could be seen occasionally in individual pigs during the entire period studied.

**Table 4:** Mean levels of Serum amyloid A (SAA) in serum (mg per L).

Day after arrival	PMWS pigs	Healthy pigs, low level of maternal antibodies	Healthy pigs, high level of maternal antibodies
	(n = 4)	(n = 17)	(n = 17)
Day 9	424 ± 475	148 ± 306	115 ± 250
Day 17	24 ± 17	27 ± 33	57 ± 76
Day 23	111 ± 114	134 ± 304	94 ± 134
Day 34	39 ± 26	57 ± 62 *	17 ± 8 *
Day 43	92 ± 120	71 ± 109	31 ± 41
Day 55	-	38 ± 75	42 ± 87

Mean values within day indicated with *differ (p < 0.05) from each other

All pigs that developed PMWS were dead at day 55. Pigs without clinical signs of PMWS were grouped as having low or high maternal immunity to PCV2. All these pigs were alive at day 55

## Discussion

The close examination of 40 randomly selected pigs suggests that four pigs denoted as "thin" actually developed PMWS. This was confirmed by necropsies in three of them, and necropsy still is the golden standard for diagnosing PMWS in individual pigs [1-3,26]. It is notable that all PMWS-affected pigs had low levels of maternal antibodies to PCV2, and that none out of the 17 pigs with high levels of maternal antibodies to PCV2 developed clinical signs resembling PMWS. This concurs well with suggestions that antibodies to PCV2 can hinder the development of PMWS [30-33]. The IPMA-method used in this study does not measure truly neutralising antibodies, but a positive correlation between neutralising antibodies and total amount of antibodies has previously been reported [34,35].

Results from the present study support the important role of the maternal immunity in preventing development of PMWS, as also suggested by others [31,36]. However, Table 1 shows that every nucleus herd sending pigs to the station had delivered individual pigs with low levels of maternally derived antibodies to PCV2, *i.e*. pigs that potentially could develop PMWS but did not. This is consistent with an earlier report showing that some farm pigs with low levels of PCV2 antibodies in serum did not develop PMWS whereas some pigs with higher levels did [34]. The present study confirms this finding and suggests that low levels of maternal antibodies to PCV2 in piglets do not necessarily lead to development of PMWS. Indeed, 17 pigs with low levels of antibodies to PCV2 on arrival remained free from PMWS. These pigs responded better to the PCV2 exposure than pigs developing PMWS in terms of a rapid development of antibodies to PCV2. Pigs that developed PMWS basically did not seroconvert to PCV2 as they became diseased. The absence of a proper immune response to PCV2 in these pigs undoubtedly contributed to the excessive proliferation of PCV2 which is commonly seen in pigs affected by PMWS [25,1-3].

As stated above, every nucleus herd had sent pigs that potentially could develop PMWS to the test station. Accordingly PMWS had been diagnosed by necropsies in pigs from every nucleus herd that had delivered pigs to the test station as previously reported [37]. As clinical signs resembling PMWS significantly less often had been attended in pure bred conventional Hampshire boars (2.8%; n = 497) than in pure bred conventional Yorkshire (8.8%; n = 509) or Landrace boars (11.3; n = 655) [37], a genetic difference in resistance to development of the disease between breeds may be indicated. This has also been indicated by others, suggesting a lower resistance towards development PCV2-associated lesions of Landrace pigs [38,39]. However, the station mixed pigs from different sources and also the effect of stressors and pathogen load at the herds of origin should be taken into account. Indeed, there was a variation in the incidence of pigs with clinical signs resembling PMWS within breeds depending on the herd of origin [37].

A higher level of PCV2 genome copies in serum was recorded in pigs that developed PMWS than in pigs that remained healthy. All four PMWS-affected pigs had expressed levels well above log 7 of PCV2 per ml serum. However, serum concentrations above log 7 of PCV2 per ml were also recorded in several pigs that were not denoted as "thin" (11 out of 17 pigs with low, and in 7 out of 17 pigs with high levels of maternal antibodies), which makes detection of PCV2 virus in serum unsuitable as a single diagnostic tool to diagnose PMWS. However, as significantly lower peak levels of PCV2 were recorded in pigs with high levels of maternal antibodies, an important role of antibodies to PCV2 in preventing an excessive proliferation of the virus was again indicated [30-33,35].

It has been reported that an unrestrained growth of PCV2 in pigs with low levels of serum antibodies with concurrent infections and/or another stressor are required for development of PMWS [1-3,40]. Production of high levels of SAA in pigs can be indicative of acute bacterial infections [41], and SAA has also been reported to be increased in pigs diseased with PCV2 [42]. However, these authors compared the serum levels of several acute phase proteins in pigs of different sources and ages affected by different diseases with that of SPF pigs aged ten weeks, and it cannot be ruled out that the levels of acute phase proteins they reported could have been partly age and herd dependent [42]. Such an effect of age has previously been shown with respect to the acute phase protein haptoglobin [43]. Furthermore, individual serum levels of both pig-MAP and haptoglobin in PCV2 negative pigs could exceed that of equally aged PCV2-positive pigs in the same herd [44]. In the present study, no association between SAA levels and PCV2 viral load was detected. Instead, the concentrations of SAA peaked on day nine after arrival, mirroring the effect of mixing pigs of different origin and thereby exposing them to an unfamiliar flora of microorganisms [45,46]. Accordingly, this peak in SAA concentrations is likely to decay over time due to an adaption of the immune system to the new environmental flora [46], and acute phase proteins appears to be less valuable as indicators for PMWS.

In conclusion, the higher PVC2 viral load observed in pigs that developed PMWS agrees with suggestions of the importance of a rapid and relevant immune response in preventing PMWS [30-33]. The peak viral load was also seen earlier in pigs that developed PMWS, possibly indicating an impaired immune function in pigs developing PMWS. However, it is also of interest that a majority of the pigs with low maternally derived antibodies to PCV2 did not develop PMWS. This study was carried out in a progeny test station allocating and mixing recently weaned piglets at an early age. Thus, both the age of the pigs in relation to stressors, as well as their age at weaning, may be of importance for the development of PMWS.

## Competing interests

The authors declare that they have no competing interests.

## Authors' contributions

PW, GBe initiated in the study and deigned it in co-operation with CF, FW and GA. IMB and CMJ was responsible for the PCR-analyses and GBl for the IPMA-analysis. PW was head writer of the manuscript with help from the other autors. All authors read and approved the final manuscript.

## References

[B1] Allan GM, Ellis JA (2000). Porcine circovirus: a review. J Vet Diagn Invest.

[B2] Segalés J, Domingo M (2002). Postweaning multisystemic wasting syndrome (PMWS) in pigs. A review. VetQ.

[B3] Opriessnig T, Meng XJ, Halburg PG (2007). Porcine circovirus type 2 associated disease: update on current terminology, clinical manifestations, pathogenesis, and intervation strategies. J Vet Diagn Invest.

[B4] Timmusk S, Wallgren P, Belák K, Berg M, Fossum C, Adair B, Allan G, Todd D (2005). Genetic analysis of PCV2 capsid protein sequences reveals two main groups of Swedish isolates. Proceedings of the International Conference on Animal Circoviruses and Associated Diseases; 11–13 July; Belfast, Nothern Ireland.

[B5] Allan GM, McNeilly F, McMenamy M, McNair I, Krakowka S, Timmusk S, Walls D, Donnelly M, Minahin D, Ellis J, Wallgren P, Fossum C (2007). Temporal distribution of porcince circovirus 2 genogroups recovered from postweaning multisystemic wasting syndrome affected and nonaffected farms in Ireland and Nothern Ireland. J Vet: Diagn Invest.

[B6] Hesse R, Kerrigan M, Rowland R (2007). Evidence for recombination between PCV2a and PCV2b in the field. Virus Res.

[B7] Dupont K, Nielsen EO, Bækbo P, Larsen LE (2008). Genomic analysis of PCV2 isolates from Danish archives and current PMWS case-control study supports a shift in genotypes with time. Vet Microbiol.

[B8] Timmusk S, Wallgren P, Brunborg IM, Hasslung Wikström F, Allan GM, McMenamy M, McNeilly F, Fuxler L, Belák K, Berg M, Fossum C (2008). Sequence analysis reveals three main genogroups of PCV2 among Swedish pigs. Virus Genes.

[B9] Allan GM, Kennedy S, McNeilly F, Foster JC, Ellis JA, Krakowka SJ, Meehan BM, Adair BM (1999). Experimental reproduction of severe wasting disease by co-infection of pigs with porcine circovirus and porcine parvovirus. J Comp Path.

[B10] Allan GM, McNeilly F, Meehan B, Ellis JE, Connor TJ, McNair I, Krakowka S, Kennedy S (2000). A sequential study of experimental infections of pigs with porcine circovirus and porcine parvovirus: Immunostaining of cryostat sections and virus isolation. J Vet Med B.

[B11] Krakowka S, Ellis JA, Meehan B, Kennedy S, McNeilly F, Allan G (2000). Viral wasting syndrome of swine: experimental reproduction of postweaning multisystemic wasting syndrome in gnotobiotic swine by coinfection with porcine circovirus 2 and porcine parvovirus. Vet Path.

[B12] Rose N, Larour G, Le Diguerher G, Eveno E, Jolly JP, Blanchard P, Oger A, Le Dimna M, Jestin A, Madec F (2003). Risk factors for porcine post-weaning multisystemic wasting syndrome (PMWS) in 149 French farrow-to-finish herds. Prev Vet Med.

[B13] Ellis J, Krakowka S, Lairmore M, Haines D, Bratanich A, Clark E, Allan G, Konoby C, Hassard L, Meehan B, Martin K, Harding J, Kennedy S, McNeilly F (1999). Reproduction of lesion of postweaning multisystemic wasting syndrome in gnotobiotic piglets. J Vet Diagn Inv.

[B14] Allan GM, McNeilly F, Ellis J, Krakowka S, Meehan B, McNair I, Walker I, Kennedy S (2000). Experimental infection of colostrum deprived piglets with porcine circovirus 2 (PCV2) and porcine reproductive and respiratory syndrome virus (PRRSV) potentiates PCV2 replication. Arch Vir.

[B15] Harms PA, Sorden SD, Halbur PG, Bolin S, Lager K, Morozov I, Paul PS (2002). Experimental reproduction of severe disease in CD/CD pigs concurrently infected with type 2 porcine circovirus and PRRSV. Vet Path.

[B16] Opriessnig T, Thacker EL, Yu S, Fenaux M, Meng XJ, Halbur PJ (2004). Experimental reproduction postweaning multisystemic wasting syndrome in pigs by dual infection with Mycoplasma hyopneumoniae and porcine circovirus type 2. Vet Pathol.

[B17] Krakowka S, Ellis JA, McNeilly F, Ringler S, Rings DM, Allan G (2001). Activation of the immune system is the pivotal event in the production of wasting disease in pigs infected with porcine circovirus-2 (PCV-2). Vet Path.

[B18] Krakowka S, Ellis JA, McNeilly F, Gilpin D, Meehan BM, McGallow K, Allan G (2002). Immunologic features of porcine circovirus type 2 infection. Vir immunol.

[B19] Ballasch M, Segalés J, Rosell C, Domingo M, Mankertz A, Urniza A, Plana-Duran J (1999). Experimental inoculation of conventional pigs with tissue homogenates from pigs with post-weaning multisystemic wasting syndrome. J Comp Path.

[B20] Albina E, Truong C, Hutet E, Blanchard P, Cariolet R, L'Hospitalier R, Mahé D, Allée C, Morvan H, Amenna N, Le Dimna M, Madec F, Jestin A (2001). An experimental model for post-weaning multisystemic wasting syndrome (PMWS) in growing piglets. J Comp Path.

[B21] Kristensen CS, Bille Hansen V, Vigre H, Bøtner A, Bækbo P, Enøe C, Larsen L, Nielsen JP, Jorsal SE (2006). Transmission of PMWS between penmates. Proceedings of the 19th International pig Veterinary Society Congress; 16–19 July; Copenhagen, Denmark.

[B22] Enøe C, Vigre H, Nielsen EO, Bøtner A, Bille-Hansen V, Jorsal SE, Bækbo P, Nielsen JP, Jorsal SE (2006). A Danish case-control study on risk factors for PMWS – Biosecurity in the herd. Proceedings of the 19th International pig Veterinary Society Congress; 16–19 July; Copenhagen, Denmark.

[B23] Wallgren P, Hasslung F, Bergström G, Linder A, Belák K, Hård af Segerstad C, Stampe M, Molander B, Björnberg Kallay T, Nörregård E, Ehlorsson CJ, Thörnquist M, Fossum C, Allan GM, Robertsson JÅ (2004). Postweaning multisystemic wasting syndrome – PMWS. The first year with the disease in Sweden. Vet Q.

[B24] Holmgren N, Lundeheim N (2002). Development of rearing systems and health for fattening pigs in Sweden. Sv Vet Tidn.

[B25] Sorden SD (2000). Update on porcine circovirus and postweaning multisystemic wasting syndrome. Swine health Prod.

[B26] Segalés J, Allan GM, Domingo M (2005). Porcine circovirus diseases. Anim Health Res Rev.

[B27] Brunborg IM, Jonassen CM, Moldal T, Bratberg B, Lium B, Koenen F, Schönheit J (2007). Association of myocarditis with high viral load of porcine circovirus type 2 in several tissues in cases of fetal death and high mortality in piglets. A case study. J Vet Diagn Invest.

[B28] Ladekjaer-Mikkelsen AS, Nielsen J, Stadejek T, Dtoorgaard T, Krakowka S, Ellis J, McNeilly F, Allan G, Bötner A (2002). Reproduction of postweaning multisystemic wasting syndrome (PMWS) in immunostimulated and non-immunostimulated 3-week-old piglets experimentally infected with porcine circovirus type 2 (PCV2). Vet Microbiol.

[B29] Ellis J, Hassard L, Clark E, Harding J, Allan G, Wilson P, Strokappe J, Martin K, McNelly F, Meehan B, Todd D, Haimes D (1998). Isolation of circovirus from lesions of pigs with postweaning multisystemic wasting syndrome. Can Vet J.

[B30] Blanchard P, Mahé D, Cariolet R, Keranaflech A, Baudoard MA, Cordoli P, Albina E, Jestin A (2003). Protection of swine against post-weaing wasting syndrome (PMWS) by porcine circovirus type 2 (PCV2) proteins. Vaccine.

[B31] McKeown NE, Opriessnig T, Thomas P, Gunette DK, Elvinger F, Fenaux M, Halbur PG, Meng XJ (2005). Effects of porcine circovirus type 2 (PCV2) maternal antibodies on experimental infection of piglets with PCV2. Clin Diagn Lab Immunol.

[B32] Meerts P, Van Gucht S, Cox E, Vandebosch A, Nauwynck HJ (2005). Correlation between type of adaptive immune response against porcine circovirus type 2 and level of viral replication. Viral Immunol.

[B33] Meerts P, Misinzo L, Lefebre D, Nielsen J, Bötner A, Kristensen CS, Nauwynck HJ (2006). Correlation between the presence of neutralizing antibodies against porcine circovirus 2 (PCV2) and protection against replication of the virus and development of PCV2-associated disease. BMC Vet Res.

[B34] McNeilly F, McNair I, Stewart G, Allan G, Green LE, Waldner C, Ellis J, Armstrong D, Krakowka S (2006). Post-weaning multisystemic wasting syndrome: Studies on disease progress in relation to serum antibody levels to porcine circovirus type 2 (PCV2) in sows and piglets and PCV2 viraemia in young pigs. Pig J.

[B35] Fort M, Olvera A, Sibila M, Segalés J, Mateu E (2007). Detection of neutralizing antibodies in post weaning multisystemic wasting syndrome (PMWS)-affected and non PMWS-affected pigs. Vet Microbiol.

[B36] Ostanello F, Caprioli A, Di Francesco A, Battilani M, Sala G, Sarli G, Mandrioli L, McNeilly F, Allan GM, Prosperi S (2005). Experimental infection of 3-week old conventional colostrums-fed pigs with porcine circovirus type 2 and porcine parvovirus. Vet Microbiol.

[B37] Bergström G, Wallgren P, Nielsen JP, Jorsal SE (2006). The incidence of thin-to-wasting pigs with respect to race in a progeny test station affected by PMWS. Proceedings of the 19th International pig Veterinary Society Congress; 16–19 July; Copenhagen, Denmark.

[B38] Opreissnig T, Fenaux M, Thomas P, Hoogland MJ, Rotschild MF, Meng XJ, Halbur PG (2006). Evidence of breed-dependant differences in susceptibility to porcine circovirus type 2-associated diseases and lesions. Vet Pathol.

[B39] Opriessnig T, Patterson AR, Madson DM, Pal N, Rotschild M, Kuhar D, Lunnev JK, Juhan NM, Meng XJ, Halbur PG (2009). Difference in severity of porcine circovirus type 2 (PCV2)-induced pathological lesions between Landrace and Pietrain pigs. J anim Sci.

[B40] Wallgren P, Belák K, Ehlorsson CJ, Bergström G, Lindberg M, Fossum C, Allan GM, Robertsson JÅ (2007). Post Weaning Multisystemic Wasting syndrome in Sweden: From an exotic to an endemic disease!. VetQ.

[B41] Hultén C, Johansson E, Fossum C, Wallgren P (2003). Interleukin 6, serum amyloid A and haptoglobin as markers of treatment efficacy in pigs experimentally infected with *Actinobacillus pleuropneumoniae*. Vet Microbiol.

[B42] Para MD, Fuentes P, Tecles F, Martínez-Subiela S, Martínez JS, Muños A, Cerón JJ (2006). Porcine acute phase protein concentration in different diseases in fiels conditions. J Vet Med B.

[B43] Petersen HH, Ersbøl AK, Jensen CS, Nielsen JP (2002). Serum haptoglobin concentrations in Danish slaughter pigs of different health status. Prev Vet Med.

[B44] Segalés J, Piñeiro C, Lampreave F, Norfarías M, Mateu E, Calsamiglia M, Andrés M, Morales J, Piñeiro M, Domingo M (2004). Haptoglobin and pig-major acute phase protein are increased in pigs with post weaning multisystemic wasting syndrome (PMWS). Vet Res.

[B45] Artursson K, Wallgren P, Alm GV (1989). Appearance of interferon-α in serum and signs of reduced immune functions in pigs after transport and installation in a fattening farm. Vet Immunol Immunopath.

[B46] Wallgren P, Artursson K, Fossum C, Alm GV (1993). Incidence of infections in pigs bred for slaughter revealed by elevated serum levels of interferon and development of antibodies to *Mycoplasma hyopneumoniae *and *Actinobacillus pleuropneumoniae*. J Vet Med B.

